# The Transcription Factor *FEZF1*, a Direct Target of *EWSR1-FLI1* in Ewing Sarcoma Cells, Regulates the Expression of Neural-Specific Genes

**DOI:** 10.3390/cancers13225668

**Published:** 2021-11-12

**Authors:** Laura García-García, Enrique Fernández-Tabanera, Saint T. Cervera, Raquel M. Melero-Fernández de Mera, Santiago Josa, Laura González-González, Carlos Rodríguez-Martín, Thomas G. P. Grünewald, Javier Alonso

**Affiliations:** 1 Unidad de Tumores Sólidos Infantiles, Instituto de Investigación de Enfermedades Raras (IIER), Instituto de Salud Carlos III (ISCIII), 28220 Madrid, Spain; laura.garciag@externos.ciemat.es (L.G.-G.); efernandezt@isciii.es (E.F.-T.); scervera@isciii.es (S.T.C.); raquel.melero@externos.isciii.es (R.M.M.-F.d.M.); santiago.josa@isciii.es (S.J.); laura.gonzalez@ciberes.org (L.G.-G.); carlosrm@isciii.es (C.R.-M.); 2 Centro de Investigación, Biomédica en Red de Enfermedades Raras, Instituto de Salud Carlos III, 28029 Madrid, Spain; 3 Division of Translational Pediatric Sarcoma Research, German Cancer Research Center (DKFZ), German Cancer Consortium (DKTK), 69120 Heidelberg, Germany; t.gruenewald@kitz-heidelberg.de; 4 Hopp-Children’s Cancer Center (KiTZ), 69120 Heidelberg, Germany; 5 Institute of Pathology, Heidelberg University Hospital, 69120 Heidelberg, Germany

**Keywords:** Ewing sarcoma, EWSR1-FLI1, GGAA-microsatellites, FEZF11

## Abstract

**Simple Summary:**

Ewing sarcoma is a rare pediatric tumor characterized by chromosomal translocations that give rise to aberrant chimeric transcription factors (e.g., EWSR1-FLI1). EWSR1-FLI1 defines a specific transcriptomic profile in Ewing sarcoma cells, which determines the tumorigenesis process. Our study focused on the identification of transcription factors regulated by EWSR1-FLI1. FEZF1 (FEZ family zinc finger protein 1), a transcription factor involved in neural cell identity, was identified as one of the most strongly upregulated genes by EWSR1-FLI1. Functional studies were carried out to characterize the involvement of FEZF1 in Ewing sarcoma pathogenesis. As a result, the inhibition of FEZF1 diminished clonogenicity and cell proliferation in three Ewing sarcoma cell lines. Transcriptomic analysis revealed several neural-specific genes transcriptionally regulated by FEZF1 and concomitantly regulated by EWSR1-FLI1, which could explain the neural-like phenotype observed in several Ewing sarcoma cell lines and tumors.

**Abstract:**

Ewing sarcoma is a rare pediatric tumor characterized by chromosomal translocations that give rise to aberrant chimeric transcription factors (e.g., EWSR1-FLI1). EWSR1-FLI1 promotes a specific cellular transcriptional program. Therefore, the study of EWSR1-FLI1 target genes is important to identify critical pathways involved in Ewing sarcoma tumorigenesis. In this work, we focused on the transcription factors regulated by EWSR1-FLI1 in Ewing sarcoma. Transcriptomic analysis of the Ewing sarcoma cell line A673 indicated that one of the genes more strongly upregulated by EWSR1-FLI1 was FEZF1 (FEZ family zinc finger protein 1), a transcriptional repressor involved in neural cell identity. The functional characterization of FEZF1 was performed in three Ewing sarcoma cell lines (A673, SK-N-MC, SK-ES-1) through an shRNA-directed silencing approach. FEZF1 knockdown inhibited clonogenicity and cell proliferation. Finally, the analysis of the FEZF1-dependent expression profile in A673 cells showed several neural genes regulated by FEZF1 and concomitantly regulated by EWSR1-FLI1. In summary, FEZF1 is transcriptionally regulated by EWSR1-FLI1 in Ewing sarcoma cells and is involved in the regulation of neural-specific genes, which could explain the neural-like phenotype observed in several Ewing sarcoma tumors and cell lines.

## 1. Introduction

Ewing sarcoma is an aggressive cancer affecting children and young adults. Ewing sarcoma cells are highly undifferentiated, and although tumors initially respond well to chemotherapy and radiotherapy, local and distant relapses are frequent [1]. From a molecular point of view, Ewing sarcoma is characterized by chromosomal translocations that give rise to chimeric proteins that act as aberrant transcription factors. The most frequent chromosomal translocation is t (11;22), which produces an aberrant transcription factor formed by the fusion of the *EWSR1* gene with the ETS transcription factor *FLI1*. The resulting aberrant transcription factor EWSR1-FLI1 affects the expression, directly or indirectly, of hundreds of genes that globally induce cell proliferation and block cell differentiation [2]. Since these chimeric transcription factors are present in all Ewing sarcoma tumors, it is widely accepted that they are the main oncogenic driver in Ewing sarcoma [1].

EWSR1-FLI1 is able to bind directly to the promoters of its target genes in two different ways. On the one hand, EWSR1-FLI1 can recognize canonical ETS response elements in the promoters of target genes. Interestingly, when EWSR1-FLI1 binds to such promoters, it inhibits transcription of the target gene instead of activating it [3]. On the other hand, EWSR1-FLI1 binds to GGAA-microsatellites localized in gene promoters or intergenic regions, where they are converted by EWSR1-FLI1 binding into de novo enhancers. In these cases, EWSR1-FLI1 strongly actives the transcription of target genes [3]. EWSR1-FLI1 can also regulate the expression of other genes indirectly, for example, by regulating the expression of other transcription factors or transcriptional (co)regulators. Two good examples of this are *NR0B1* and *NKX2-2* [4,5,6]; both genes, which encode transcriptional repressors, are positively induced by EWSR1-FLI1. In consequence, the gene expression profile depending on EWSR1-FLI1 is partially dependent on other transcriptional regulators. Thus, EWSR1-FLI1 is placed at the summit of a complex network of hierarchized transcriptional regulations that ultimately produce the oncogenic expression profile.

Although Ewing sarcoma was described by James Ewing one hundred years ago [6], and arises in 85% of cases in bones, there are still controversies about the origin cells. Experimental data, mainly based on the analysis of gene expression profiles regulated by EWSR1-FLI1 in different cell contexts, suggest that Ewing sarcoma probably arises from mesenchymal cell lineages resident in bones. Thus, EWSR1-FLI1 knock-down was able to induce the expression of gene expression profiles characteristic of a different mesodermal lineage [7]. However, other studies suggest that Ewing sarcoma maintains some properties characteristic of cells from the neuro-ectodermal lineage. In fact, it is well known that some Ewing sarcoma tumors and cell lines display neuro-ectodermal characteristics [8,9,10,11], which are more clearly displayed in response to certain stimuli [12,13]. Additionally, transcriptomic and functional analyses support the existence of a residual neural-like phenotype in Ewing sarcoma [14,15,16].

Deciphering the transcriptional regulators that are under EWSR1-FLI1 control is relevant to understanding the biology of Ewing sarcoma and identifying pathways that could participate in Ewing sarcoma pathogenesis [17,18]. With this in mind, we looked for transcriptional regulators that were positively or negatively regulated by EWSR1-FLI1 in the Ewing sarcoma cell line A673 using the well-established cell model A673/TR/shEF in which EWSR1-FLI1 is downregulated by a specific doxycycline-dependent shRNA [19]. FEZF1/ZNF312B (FEZ family zinc finger protein 1/Zinc finger protein 312B) was identified as the transcriptional regulator most positively upregulated by EWSR1-FLI1.

FEZF1 is a highly conserved transcription factor belonging to the large family of C2H2 zinc finger proteins [20,21,22]. FEZF1 is expressed early during mouse development and is important for brain development and cell identity [23,24]. Particularly, FEZF1 is involved in axonal projection and proper termination of olfactory sensory neurons, as evidenced by the fact that in FEZF1 knock-out mice, olfactory neurons fail to mature [24]. Beyond its role in brain development, little is known about the involvement of FEZF1 in disease, although some studies suggest a role of FEZF1 in cancer. Thus, FEZF1 knockdown reduced cell proliferation and migration in human cervical cancer cell lines and was shown to be an independent predictive factor for recurrence in cervical cancer [25].

In this work, we showed that FEZF1 is highly expressed in Ewing sarcoma cells and positively upregulated by EWSR1-FLI1. FEZF1 knock-down in three independent Ewing sarcoma cell lines reduced cell proliferation. Finally, we show that FEZF1 regulates the expression of a group of neural genes that include some of the genes regulated by EWSR1-FLI1. We concluded that FEZF1 is a direct target of EWSR1-FLI1 in Ewing sarcoma and that FEZF1 could be involved in the neural-like phenotype observed in some Ewing sarcoma tumors by regulating a subset of neural-specific genes.

## 2. Materials and Methods

### 2.1. Cell Lines

A673/TR/shEF cells, which express a specific shRNA inducible by doxycycline directed against EWSR1-FLI1 mRNA, have been previously described in detail [19]. A673/TR/shEF cells were maintained in DMEM supplemented with 10% tetracycline-free FBS (Capricorn Scientific, Ebsdorfergrund, Germany), 50 U/mL penicillin, 50 µg/mL, 100 μg/mL zeocin and 5 μg/mL blasticidin. A673/TR/shEF cells were stimulated with doxycycline (1 µg/mL) (Formedium, Norfolk, UK) to induce the expression of EWSR1-FLI1 specific shRNA. A673, U2-OS and SAOS-2 cell lines were cultivated in DMEM. SK-N-MC cell line was maintained in DMEM supplemented with 1xMEM non-essential amino acids. A4573, CADO-ES-1, MHH-ES-1, RD-ES, and CAL-78 cell lines were maintained in RPMI 1640 medium. SK-PN-DW and TC-71 were cultured in IMDM and SK-ES-1 in McCoy’s medium. CAL-72 cell line was maintained in DMEM supplemented with 1x insulin transferrin sodium selenite (Merck Life Science, Darmstadt, Germany). All media were supplemented with 10–20% FBS, penicillin and streptomycin. All cells were periodically tested for mycoplasma contamination (Mycoalert mycoplasma detection kit, #LT07-318, Lonza, Basel, Switzerland) and were authenticated by STR profiling at the Genomic Facility at Biomedical Research Institute (IIB-CSIC, Madrid, Spain).

### 2.2. Establishment of EwingSarcoma Cell Lines Expressing Doxycycline-Inducible FEZF1 shRNA

BLOCK-iT lentiviral expression system (Invitrogen, Waltham, MA, USA) was used to establish Ewing sarcoma cell lines harboring doxycycline-inducible small hairpin RNAs (shRNA) against FEZF1, as previously described [19]. Ewing sarcoma cell lines A673, SK-N-MC and SK-ES-1 were infected with lentiviruses containing the pLenti6/TR expression plasmid (Invitrogen) to establish stable cell lines expressing constitutively the tetracycline repressor (TR). One clone for each cell line expressing the highest levels of tetracycline repressor, as assayed by western-blot (designed A673/TR, SK-N-MC/TR and SK-ES-1/TR), was chosen for the next steps. Target shRNA sequence for FEZF1 was designed using the BLOCK-iT RNAi Designer web application (Invitrogen). Complementary oligonucleotide sequences, located between nucleotides 725–745 of the FEZF1 mRNA sequence (GenBank accession number: NM_001160264.2) were annealed and inserted into the pENTR-BLOCK-iT plasmid (Invitrogen), and afterward, the H1/shRNA cassette was transferred by recombination to the pLenti4-BLOCK-iT plasmid according to the manufacturer’s instructions (Invitrogen). The sequence of the oligonucleotides was as follows: shFEZF1 (forward), GATCCCGGTCTTTAATGCGCACTATAATTCAAGAG ATTATAGTGCGCATTAAAGACCTTTTTC; shFEZF1 (reverse), TCGAGAAAAAGGTCTTTAATGCGCACTATAATCTCTTGAATTATAGTGCGCATTAAAGACCGG. Subsequently, 1 × 10^6^ cells were transfected by electroporation with 10 µg pLenti4/H1/shFEZF1 lentiviral vector DNA in 100 µL Opti-MEM medium) using a NEPA-21 electroporator (Nepa Gene, Ichikawa, Japan). Electroporation parameters were, for poring pulse: 150 V (SK-ES-1 and A673) or 125V (SK-N-MC), 5 ms pulse length, 50 ms pulse interval, 2 cycles, decay rate 10%, polarity +; for transfer pulse: 20 V, 50 ms pulse length, 50 ms pulse interval, 5 cycles, decay rate 40%, polarity +/−. After electroporation, cells were maintained in standard conditions for 24 h to allow recovery and then selected with zeocin (100 µg/mL). Stable clones were checked for FEZF1 knockdown by RT-qPCR and western-blot after 72-h stimulation with doxycycline (1 µg/mL). Clones showing the highest levels of protein inhibition upon doxycycline stimulation were selected for additional studies.

### 2.3. Western Blot Analysis and Antibodies

The procedure is described in detail elsewhere [26]. Primary antibodies were as follows: anti-FLI1 rabbit monoclonal antibody from Abcam (#ab133485) (Cambridge, UK), anti-FEZF1 mouse monoclonal antibody from Santa Cruz Biotechnology (#sc-515487) (Dallas, TX, USA), and HRP-anti-α-tubulin antibody from Abcam (#ab185067) (St. Louis, MO, USA). Goat anti-mouse (#sc-2055) and goat anti-rabbit IgG (#sc-2054) horseradish peroxidase-conjugated secondary antibodies were purchased from Santa Cruz Biotechnology.

### 2.4. Immunofluorescence

A673/TR/shEF cells were seeded at 4000 cells on glass cover slides in 24-well plates and stimulated with doxycycline (1 µg/mL) for 72 h. Then, cells were washed in PBS, fixed in 4% paraformaldehyde for 15 min, and permeabilized with 0.1% Triton X-100. After that, cells were incubated overnight with the primary antibody at 4 °C (anti-FEZF1 diluted in 4% FBS in PBS), washed, and incubated with secondary antibody for 1.5 h at room temperature (anti-mouse Alexa Fluor 488 conjugated). Cells were counterstained with 4,6-diamidino-2-phenylindole (DAPI), washed 4 × 10 min with PBS and mounted on slides using ProLong^™^ Gold antifade mounting medium (#P36934, Thermo Fisher Scientific, Waltham, MA, USA). Cells were visualized in a fluorescence microscope (Leica, Wetzlar, Germany).

### 2.5. Bromodeoxyuridine Proliferation Assay

Cells were seeded in 96 multi-well plates (2500–3500 cells/well, 8-replicates) and cultured with or without doxycycline for 72 h (1 µg/mL). The incorporation of bromodeoxyuridine into DNA was quantified using a chemiluminescent assay (Roche, Basel, Switzerland). Chemiluminescence was quantified using a microplate reader (Infinite M200, Tecan, Mannerdorf, Switzerland).

### 2.6. Clonogenic Assay

A673/TR/shFEZF1, SK-ES-1/TR/shFEZF1 and SK-N-MC/TR/shFEZF1 cells were plated in triplicate in a 24-well plate at 1 × 10^3^, 2 × 10^3^ or 4 × 10^3^ cells per well. Thereafter, they were treated with or without doxycycline (1 μg/mL) for 9–10 days in culture medium, supplemented with 10% or 20% (SK-ES-1 cells) tetracycline-free FBS. Culture medium containing fresh doxycycline, where appropriate, was changed every 3 days. At the end of the experiment, colonies were first fixed and then stained with crystal violet and photographed.

### 2.7. Proliferation Curve Assay

A673/TR/shFEZF1, SK-ES-1/TR/shFEZF1 and SK-N-MC/TR/shFEZF1 cells were maintained in culture medium supplemented with 10% or 20% (SK-ES-1 cells) tetracycline-free FBS, with or without doxycycline (1 μg/mL) for 30–34 days. When cells reached 70–80% confluence, were trypsinized, counted and re-plated in a new *p* 100 plate and so on until completing 30–34 days. The culture medium containing fresh doxycycline was changed every 3 days when necessary. The number of population doublings was calculated with the formula: n° of population doublings = log2 (numbers of cells at the initial time/number of cells at the final time). Cell doubling time was calculated in each cycle of cell seeding—trypsinization as cell doubling time = time elapsed between cycles/n° cell population doubling observed in this period of time.

### 2.8. Multiplex Real-Time Quantitative RT-PCR (RT-qPCR)

RT-qPCR conditions, including primers and Taqman probes for EWSR1-FLI1 and TBP are described elsewhere [19,26]. TaqMan probes for FEZF1 (hs03987877_g1) and FEZF1-AS1 (hs00935566_m1) were purchased from Life Technologies (San Diego, CA, USA). Reactions were run on a RotorGene 6000 (Qiagen, Hilden, Germany). Cycle threshold (Ct) for each gene and TBP was calculated using the Rotor Gene Q Software (v.2.3.1, Qiagen, Hilden, Germany). Relative expression for each gene was calculated as 2^−^^ΔCt^, where ∆Ct = Ct_gene_ − Ct_TBP_.

For the analysis of FEZF1 expression in control normal tissues, the FirstChoice^®^ Human Total RNA Survey Panel (Applied Biosystems, Waltham, MA, USA) was used. This panel is made up of total RNA pools from 20 different human normal tissues. Each pool consists of RNA from at least 3 tissue donors.

### 2.9. Transcriptomic Analysis (RNAseq)

RNA was extracted using TRI-REAGENT according to the manufacturer’s protocol (Sigma-Aldrich, Sant Louis, MI, USA) and additionally purified using an RNeasy Mini Elute Cleanup kit (Qiagen). RNAseq was perfomed at CNAG (Centro Nacional de Análisis Genómico, Barcelona, Spain). The mRNA library was obtained with the TruSeq Stranded mRNA Library Prep Kit (Illumina, San Diego, CA, USA), and paired-end sequencing (2 × 50 pb) was carried out in a NovaSeq 6000 (Illumina). On average, 39.9 × 10^6^ reads (range 30.8–48.4 × 10^6^ reads) were obtained per sample. The average percentage of aligned reads to a single location in the reference genome ranged from 61.8% to 64.8%. Data analysis was carried out using Galaxy (usegalaxy.org), an open-source, web-based platform that integrates many tools for data-intensive biomedical research [27]. Briefly, mapping and transcript quantification were performed with Salmon quant script (v0.14.1.2), using GRCh38 as transcriptome reference. Differences in expressed features from quantification tables were calculated with DESeq2 (v2.11.40.6). Genes whose *p*-value (FDR) was <0.05 were considered differentially expressed between two experimental conditions. Gene ontology functional annotation was carried out with PANTHER [28,29].

### 2.10. Analysis of ChIP-Seq (Chromatin Immunoprecipitation Followed by Sequencing) Data

ChIP-seq data publicly available were retrieved from Gene Expression Omnibus (GEO accession number GSE61944 and GSE176400) [3,30] and displayed in the UCSC browser (genome.ucsc.edu).

Samples from GSE61944 correspond to the following accession numbers: GSM1517568_A673.WCE, GSM1517569_A673.shGFP48.FLI1, GSM1517570_A673.shGFP48.H3K27ac, GSM1517572_A673.shFLI148.FLI1, GSM1517573_A673.shFLI148.H3K27ac, GSM1517543_SKNMC.WCE, GSM1517546_SKNMC.shGFP96.FLI1, GSM1517547_SKNMC.shGFP96.H3K27ac, GSM1517555_SKNMC.shFLI196.FLI1, GSM1517556_SKNMC.shFLI196.H3K27ac.

Samples from GSE176400 correspond to the following accession numbers: GSM5363936_A673_FLI1_ChIPSeq, GSM5364006_SKNMC_FLI1_ChIPSeq, GSM5364016_TC71_FLI1_ChIPSeq, GSM5363941_CHLA10_FLI1_ChIPSeq, GSM5363956_EW22_FLI1_ChIPSeq, GSM5363961_EW24_FLI1_ChIPSeq, GSM5363981_MIC_FLI1_ChIPSeq, GSM5363986_POE_FLI1_ChIPSeq, GSM5363996_RH1_FLI1_ChIPSeq, GSM5364011_TC32_FLI1_ChIPSeq, GSM5363971_EW7_FLI1_ChIPSeq, GSM5364001_SKES1_FLI1_ChIPSeq, GSM5363991_RDES_FLI1_ChIPSeq, GSM5363976_MHHES1_FLI1_ChIPSeq, GSM5363951_EW1_FLI1_ChIPSeq, GSM5363946_CHLA25_ERG_ChIPSeq, GSM5363966_EW3_ERG_ChIPSeq.

### 2.11. Identification of GGAA Microsatellites Inside or Next to Genes

Two scripts were developed in R to determine the presence of GGAA microsatellites bound by EWSR1-FLI1 inside or next to genes. GGAA-inside-gene script [31] is able to scan a list of genes and determines if there is a GGAA microsatellite inside the gene taken from a list of experimentally confirmed EWSR1-FLI1 ChIPseq peaks. GGAA-near-promoter script [32] scans a list of genes and, for each gene, identifies the ChIPseq peak closer to both sides (upstream and downstream) of the gene, computing the distance to the transcription start site (TSS).

The following data sources were used to run the scripts. Differentially expressed genes were obtained from A673 RNA-seq experiments. Gene location (chromosome number, start and end of the gene and strand) was extracted from BioMart using R routines. GGAA microsatellites bound by EWSR1-FLI1 were extracted from two published datasets of EWSR1-FLI1 ChIPseq studies (GEO accession number GSE61944 and GSE176400) [3,30]. Output was stored in tables. All scripts have been stored in GitHub repository.

### 2.12. Determination of the Allele Sizes Containing the GGAA-Microsatellites Located in FEZF1 Promoter

To characterize the length of the different alleles corresponding to the GGAA-microsatellite located in the FEZF1 promoter in the different cell lines, a fluorescent DNA fragment covering this region was generated by PCR with primers FEZF1-F, 5′-FAM-GTAAAACGACGGCCAGTCTCTCCTAATGCCAAGCCCAAAG and FEZF1-R, 5′-CAGGAAACAGCTATGACACACGTAGAACAGGTTAGCCGCAC. PCR fragments were run in an ABI PRISM 3100 Genetic Analyzer (Applied Biosystems, Waltham, MA, USA), and allele size was determined with Peak Scanner 1.0 (Applied Biosystems).

## 3. Results

### 3.1. EWSR1-FLI1 Regulates the Expression of a Significant Number of Transcriptional Regulators

The main objective of this study was to identify transcriptional regulators that were in turn regulated by EWSR1-FLI1 in Ewing sarcoma. As a first approximation, we looked for transcription factors that were upregulated or downregulated upon EWSR1-FLI1 knockdown in the well-characterized Ewing sarcoma cell model A673/TR/shEF. In this model, EWSR1-FLI1 can be efficiently downregulated upon doxycycline-mediated induction of a specific EWSR1-FLI1 shRNA [19]. Thus, in the absence of doxycycline, A673 Ewing sarcoma cells express high levels of EWSR1-FLI1 (EWSR1-FLI1^high^), while those in the presence of doxycycline express low levels of EWSR1-FLI1 (EWSR1-FLI1^low^).

**Figure 1:**
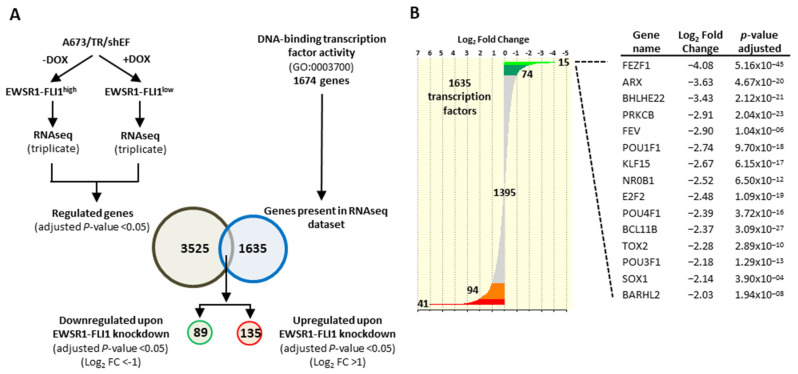
EWSR1-FLI1 regulates the expression of a significant number of transcription factors/regulators. (**A**) A673/TR/shEF cells were cultured in the absence or presence of doxycycline (DOX, 1 µg/mL, 72 h) and analyzed by RNAseq to identify EWSR1-FLI1 regulated genes. EWSR1-FLI1 regulated genes were cross-referenced with a list of transcriptional regulators obtained from the gene ontology term “DNA-binding transcription factor activity” to identify the transcriptional regulators whose expression is downregulated or upregulated upon EWSR1-FLI1 knockdown. (**B**) Distribution of 1635 transcription factors according to its expression level (Log_2_ fold change). The table shows the Log_2_ fold change and the adjusted *p*-value from the top-fifteen transcriptional regulators that were downregulated upon EWSR1-FLI1 knockdown.

Firstly, we analyzed the expression levels (RNAseq data) of a total of 1674 transcription factors that belong to the Gene Ontology term “DNA binding transcription factor activity” (GO:0003700) [33] (Table S1), in A673/TR/shEF cells incubated in the absence (EWSR1-FLI1^high^) or presence (EWSR1-FLI1^low^) of doxycycline during 72 h. A total of 1635 transcription factors were present in the RNAseq dataset. Of these, 89 transcription factors were downregulated (adjusted *p*-value ≤ 0.05, log_2_ fold change ≤ −1) while 135 were upregulated (adjusted *p*-value ≤ 0.05, log_2_ fold change ≥ 1) upon EWSR1-FLI1 knockdown (Tables S2 and S3). Thus, near 14% of the 1635 transcription factors present in the RNAseq dataset were regulated by EWSR1-FLI1 (Figure 1A). Consequently, EWSR1-FLI1 regulates the expression of a significant proportion of transcription factors, which suggests that EWSR1-FLI1 exerts its function, at least in part, through the regulation of other transcription factors. Some of these transcription factors, such as NR0B1 or BCL11B, have been previously shown to be regulated by EWSR1-FLI1 and play important functional roles in Ewing sarcoma pathogenesis [4,34,35] (Figure 1B). However, the role of many other transcription factors in Ewing sarcoma pathogenesis is absolutely unknown. The transcription factor whose expression was more strongly downregulated upon EWSR1-FLI1 knock-down was FEZF1 (Figure 1B).

FEZF1 is a critical transcription factor in nervous system development that has been recently involved in cancer progression [25]. However, the role played by FEZF1 in Ewing sarcoma has not been explored until today.

### 3.2. FEZF1 Is Upregulated by EWSR1-FLI1 and Is Highly Expressed in Ewing Sarcoma Cell Lines

**Figure 2:**
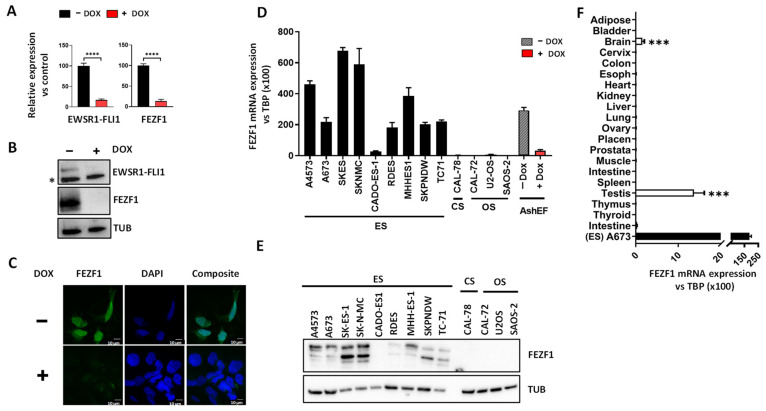
FEZF1 expression correlates positively with EWSR1-FLI1 levels and is highly expressed in Ewing sarcoma cell lines. (**A**) FEZF1 mRNA expression (RT-qPCR) in A673/TR/shEF cells stimulated with doxycycline (DOX, 1 µg/mL, 72 h). FEZF1 mRNA levels decreased more than 80% upon EWSR1-FLI1 knockdown (mean ± SD) (**B**) Western-blot analysis of FEZF1 and EWSR1-FLI1 in A673/TR/shEF cells confirm downregulation of FEZF1 protein upon EWSR1-FLI1 knock-down (asterisk denotes an unspecific band). (**C**) A673/TR/shEF cells were cultured in the absence or presence of doxycycline and FEZF1 protein detected by immunofluorescence. FEZF1 is located in the nucleus in control cells (−DOX), but its expression is lost when cells are stimulated with doxycycline to downregulate EWSR1-FLI1 levels. Scale bar: 10 µm. (**D**) FEZF1 mRNA expression levels (RT-qPCR) in a panel of bone sarcoma cell lines including Ewing sarcoma (ES), chondrosarcoma (CS), and osteosarcoma (OS). FEZF1 was exclusively expressed in Ewing sarcoma cells. FEZF1 expression in A673/TR/shEF cells is included for comparative purposes (mean ± SD). (**E**) Western-blot analysis of FEZF1 and tubulin (TUB) in the same cell lines confirmed the expression of FEZF1 only in Ewing sarcoma cell lines. (**F**) FEZF1 mRNA levels (RT-qPCR) were analyzed using a commercial source of human RNA from normal tissues. FEZF1 is expressed specifically in the testis and brain, although even in these tissues, its expression is significantly lower than the observed in the Ewing sarcoma cell line A673 (mean ± SD). (*** *p* < 0.001, **** *p* < 0.0001; Student’s *t*-test).

Quantitative RT-PCR and western-blot confirmed the results obtained with the RNAseq data. As shown in Figure 2, expression of FEZF1 mRNA (Figure 2A) and protein (Figure 2B, Figure S4) were dramatically downregulated upon EWSR1-FLI1 knock-down in A673/TR/shEF cells. As expected, FEZF1 was mainly expressed in the nucleus of A673/TR/shEF cells (Figure 2C). Nuclear expression was notably reduced in A673/TR/shEF cells when EWSR1-FLI1 expression was downregulated.

We next compared the expression of FEZF1 in a panel of bone sarcoma cell lines, including Ewing sarcoma (*n* = 9), osteosarcoma (*n* = 3) and chondrosarcoma (*n* = 1). Ewing sarcoma cell lines expressed high levels of FEZF1 mRNA (Figure 2D) and protein (Figure 2E, Figure S4). The only exception was the Ewing sarcoma cell line CADO-ES1, in which FEZF1 was not detected. FEZF1 expression was undetectable at the mRNA and protein level in osteosarcoma and chondrosarcoma cells. This indicates that FEZF1 is highly expressed in Ewing sarcoma cells and that FEZF1 expression was specific for Ewing sarcoma, at least when compared to other bone sarcomas. To strengthen these results, we analyzed the datasets available from a study published during the preparation of this manuscript and in which the authors carried out a multi-omics approach to characterize an extensive list of Ewing sarcoma cell lines [30]. According to this study, FEZF1 is expressed at high levels in all Ewing sarcoma cell lines studied, including cells expressing EWSR1-FLI1 and EWSR1-ERG fusion proteins. In addition, FEZF1 was downregulated when the fusion proteins were knocked down (Figure S1). These results clearly demonstrate that FEZF1 is robustly regulated by EWSR1-FLI1 and EWSR1-ERG fusion proteins in Ewing sarcoma cells.

Finally, we analyzed the expression levels of *FEZF1* mRNA in a panel of normal human tissues. FEZF1 expression was highly tissue-specific. In fact, it was only expressed in the brain and testis (Figure 2F). Remarkably, the expression in such tissues compared to the *TBP* housekeeping gene was more than 10-fold lower than the expression levels observed in Ewing sarcoma cell lines.

Altogether, FEZF1 expression correlated positively with that of EWSR1-FLI1, and as a consequence, FEZF1 is highly expressed in Ewing sarcoma cells. Interestingly, FEZF1 expression was also demonstrated to be tumor- and tissue-specific.

### 3.3. The FEZF1 Promoter Contains a GGAA-Microsatellite

**Figure 3:**
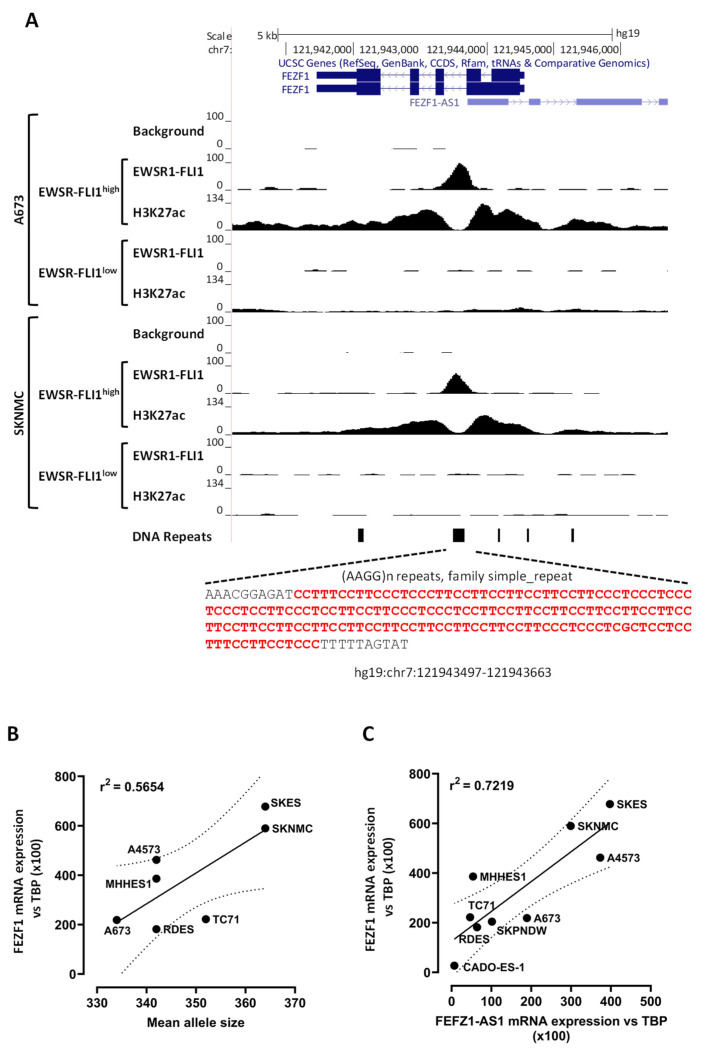
EWSR1-FLI1 binds a GGAA-microsatellite located into the *FEZF1* gene, and the number of GGAA-repeats correlates with FEZF1 expression levels. (**A**) The genomic region corresponding to *FEZF1* and *FEZF1-AS1* genes is shown (UCSC genome browser). The location of GGAA-microsatellite and its corresponding sequence is shown at the bottom of the figure. Publicly available ChIP-seq data demonstrate the binding of EWSR1-FLI1 to GGAA-microsatellite in two different Ewing sarcoma cell lines (A673 and SK-N-MC). H3K27ac ChIP-seq marks indicate that this site is transcriptionally active when EWSR1-FLI1 is bound to the GGAA-microsatellite, but not when EWSR1-FLI1 is knocked-down. (**B**) The length of GGAA-microsatellite was determined by PCR and fluorescent fragment analysis. Mean allele length correlates with FEZF1 mRNA expression levels (for this analysis, CADO-ES1 was excluded). (**C**) Correlation of FEZF1 and FEZF1-AS1 mRNA expression levels in Ewing sarcoma cells.

One of the main mechanisms through which EWSR1-FLI1 upregulates gene expression is by binding to GGAA-microsatellites located in gene promoters or enhancers [3,36,37]. We, thus, look for GGAA microsatellites in or near the FEZF1 gene. A long GGAA-microsatellite was detected in the intron 1 of FEZF1 (Figure 3A) (chr7: 121943497-121943663, hg19). The microsatellite was composed of 28 GGAA-repeats in the human reference genome, 16 of which were contiguous and the rest separated for GGAG-repeats intercalated among GGAA-repeats. To determine if EWSR1-FLI1 binds to these GGAA-microsatellites, we reviewed a published ChIP-seq dataset to identify EWSR1-FLI1 DNA binding sites [3]. As shown in Figure 3A, EWSR1-FLI1 binds this DNA motif in A673 and SK-N-MC Ewing sarcoma cell lines, confirming that FEZF1 is a direct target of EWSR1-FLI1. Interestingly, H3K27ac ChIPseq marks, which denote a transcriptionally active site [38], were detected when EWSR1-FLI1 was bound to this GGAA microsatellite, but not when EWSR1-FLI1 was silenced. This EWSR1-FLI1 binding site was also observed in the ChIP-seq dataset from Orth et al. (Figure S1C). Altogether, these data strongly suggest that EWSR1-FLI1 actives FEZF1 transcription by binding a large GGAA-microsatellite located into the *FEZF1* gene.

This GGAA-microsatellite was located in the first intron of *FEZF1*, which can be considered an unusual location for a transcriptional activator. Thus, we analyzed how frequently GGAA-microsatellites were located in the introns of the genes regulated positively by EWSR1-FLI1. Using an in-house script, we determined that 180 out of 1456 genes positively regulated by EWSR1-FLI1 (12%) had at least one GGAA-microsatellite detected by ChIP-seq into the gene. Figure S2 shows two examples of genes regulated by EWSR1-FLI1, *PRKCB* [39] and *APCDD1* [40], in which GGAA-microsatellites were located in the introns of these genes. According to these results, it seems that the location of active GGAA-microsatellites downstream the canonical promoter of EWSR1-FLI1 target genes is not unusual and is in agreement with the fact that enhancers can be located upstream or downstream genes and also within introns [41].

Several studies have demonstrated a relationship between the number of GGAA-repeats, the affinity of EWSR1-FLI1 and the levels of mRNA observed in the target genes upregulated by EWSR1-FLI1 [4,36]. We thus analyzed the length of the FEZF1 GGAA-microsatellite in a panel of Ewing sarcoma cell lines and its correlation with *FEZF1* mRNA expression levels. As shown in Figure 3B, there was a direct correlation between the length of GGAA-microsatellite and the FEZF1 expression level.

The *FEZF1* GGAA-microsatellite is also located near *FEZF1-AS1*, a non-coding gene that is synthesized in opposite orientation to *FEZF1* [42] (Figure 3A). We thus hypothesized that FEZF1-AS1 could be regulated by EWSR1-FLI1 in Ewing sarcoma cells in a similar fashion as FEZF1. In fact, the expression levels of FEZF1-AS1 strongly correlated with FEZF1 expression in Ewing sarcoma cells, suggesting that FEZF1 and FEZF1-AS1 are coordinately regulated by EWSR1-FLI1 in Ewing sarcoma cells (Figure 3C). Thus, FEZF1-AS1 expression was also dramatically downregulated upon EWSR1-FLI1 knockdown in A673 cells, as shown in Figure S3.

### 3.4. FEZF1 Knock-Down Impairs Ewing Sarcoma Cell Proliferation

**Figure 4:**
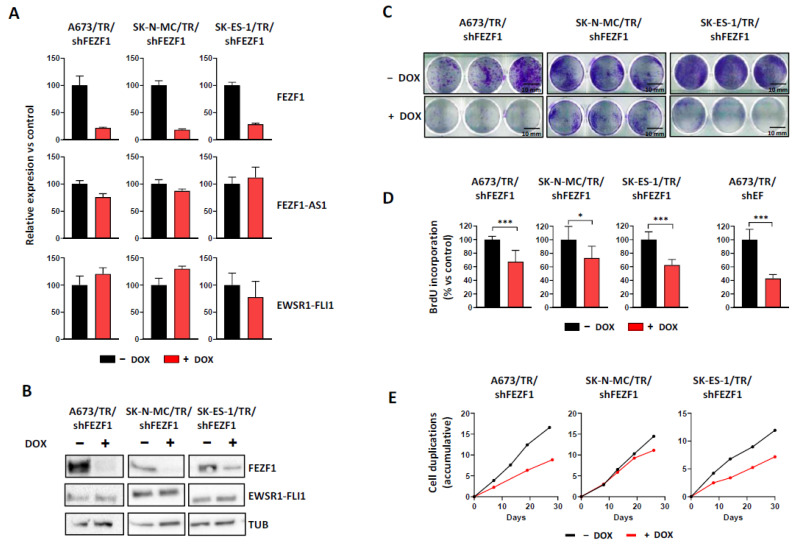
FEZF1 knockdown impairs Ewing sarcoma cell proliferation. (**A**) mRNA expression levels of FEZF1, FEZF1-AS1 and EWSR1-FLI1 in three Ewing sarcoma cell lines expressing a doxycycline-inducible shRNA directed against FEZF1 mRNA. Cells were cultured for 72 h in the absence or presence of doxycycline (1 µg/mL), and mRNA expression levels were quantified by RT-qPCR (mean ± SD). (**B**) Western-blot analysis of FEZF1 and EWSR1-FLI1 in the same cell lines, confirming the downregulation of FEZF1. EWSR1-FLI1 protein levels were not affected by FEZF1 knockdown. (**C**) Cell colony formation assays show a reduction in the number of cell colonies formed upon FEZF1 knockdown. Scale bar: 10 mm. (**D**) The number of cell duplications accumulated over 30 days in which cells were cultured in the absence or presence of doxycycline. (**E**) Cells were cultured in the absence or presence of doxycycline (1 µg/mL), and DNA synthesis was analyzed by BrdU incorporation assay. FEZF1 knock-down significantly reduced DNA synthesis in the three Ewing sarcoma cell lines analyzed. For comparative purposes, the effect of EWSR1-FLI1 knock-down in A673/TR/shEF cells is also shown (mean ± SD of one experiment out of four with equivalent results; * *p* < 0.05, *** *p* < 0.001; Student’s *t*-test).

Next, we analyzed the effect of FEZF1 on cell proliferation in Ewing sarcoma cell lines. For this, we generate three different Ewing sarcoma cell lines (A673, SK-N-MC and SK-ES-1) expressing a doxycycline-inducible shRNA directed against FEZF1 mRNA. Induction of FEZF1 shRNA with doxycycline downregulated significantly FEZF1 mRNA (Figure 4A) and protein (Figure 4B and Figure S4). Since FEZF1 and FEZF1-AS1 expression correlated strongly (Figure 3C), we analyzed if FEZF1 downregulation affected FEZF1-AS1 expression. As shown in Figure 4A, the downregulation of FEZF1 mRNA did not affect the levels of FEZF1-AS1 mRNA. In addition, EWSR1-FLI1 levels were not affected by FEZF1 downregulation (Figure 4A,B).

Once confirmed that FEZF1 knockdown did not affect EWSR1-FLI1 and FEZF1-AS1 expression, we analyzed the effect of FEZF1 downregulation on colony formation as a measure of its oncogenic potential. As shown in Figure 4C, FEZF1 knock-down significantly reduced the number of colonies formed when tumor cells were cultured at low density. This effect was observed in the three Ewing sarcoma cell lines, although the effects of FEZF1 knock-down were greater in A673 and SK-ES-1 cells compared to SK-N-MC cells. Next, we analyzed the effect of FEZF1 knock-down on cell proliferation. Firstly, we quantified the number of cell duplications accumulated during a determined time in which cells were continuously cultured in the absence or presence of doxycycline to the knock-down FEZF1 expression. As shown in Figure 4E, FEZF1 knock-down significantly reduced the number of cell duplications in A673 and SK-ES-1 cells, while the effect of FEZF1 knock-down in SK-N-MC was more modest.

To confirm the effect of FEZF1 knock-down on cell proliferation, we analyzed DNA synthesis by using a bromodeoxyuridine (BrdU) incorporation assay. As we can be observed in Figure 4D, BrdU incorporation was reduced significantly in all three Ewing sarcoma cell lines upon FEZF1 knock-down (range 27–38%), although again, the effect on SK-N-MC was smaller, in line with the previous results. Figure 4E also includes a graph showing the effect of EWSR1-FLI1 knock-down on BrdU incorporation in the A673/TR/shEF cell line. EWSR1-FLI1 knock-down produced a 57% reduction in BrdU incorporation, greater than that observed after FEZF1 knock-down. Taken together, these results suggested that FEZF1 is involved in promoting cell proliferation in Ewing sarcoma cell lines. Interestingly, the effects of FEZF1 on cell proliferation were observed even in the presence of EWSR1-FLI1 expression (Figure 4A), suggesting that FEZF1 is a relevant downstream gene target in Ewing sarcoma oncogenesis.

### 3.5. FEZF1 Regulates the Expression of a Subset of Genes Characteristic of Neural Cells

In its physiological setting, FEZF1 acts as a transcriptional repressor that plays a critical role in nervous system development [21,24]. Thus, we analyzed the gene expression profile induced upon FEZF1 silencing in the Ewing sarcoma cell line A673 in order to identify genes and functional pathways regulated by FEZF1 in Ewing sarcoma. To overcome technical and biological variability, we performed RNAseq experiments in three independent clones, and a polyclonal population of A673/TR/shFEZF1 cells stimulated with doxycycline by 72 h to downregulate FEZF1 expression. A subset of 174 genes (0.7% out of total) was differentially regulated upon FEZF1 knock-down (*p*-value adjusted ≤ 0.05; Table S4). We subjected this list of genes to gene-ontology (GO) enrichment analysis to identify characteristic functional pathways. The number of GO terms significantly enriched was low, probably because of the low number of genes differentially expressed upon FEZF1 knock-down. However, some interesting findings could be observed. Thus, genes classified in GO terms such as growth cone (GO:0030426) and transport vesicle membrane (GO:0030658), associated with neurotransmission and nervous system physiology, were enriched more than three times with respect to what was expected (FDR < 0.05) (Table 1 and Table S5).

Finally, we compared the gene expression profile regulated by EWSR1-FLI1 and FEZF1 in A673 Ewing sarcoma cells. Notably, 67 out of 174 genes regulated by FEZF1 (38.5%) were also regulated by EWSR1-FLI1, supporting the fact that FEZF1 is regulating a subset of the genes regulated by EWSR1-FLI1. Of this group of genes, 39 genes were differentially expressed in the same sense, that is, downregulated or upregulated by EWSR1-FLI1 and FEZF1 (24 upregulated and 15 downregulated upon EWSR1-FLI1/FEZF1 knock-down). Fisher’s exact test (*p* < 0.0045) confirmed that the number of genes that were regulated by both EWSR1-FLI1 and FEZF1 in the same sense was much higher than the number of genes that would be expected by chance alone.

Finally, we asked how many genes on this list had GGAA microsatellites bound by EWSR1-FLI1 and, in this way, determined whether these genes were directly regulated by EWSR1-FLI1 or by FEZF1. For that purpose, we searched the list of 67 genes for EWSR1-FLI1 peaks at a maximum of 10 kb upstream and downstream TSS using an in-house R script and the list of EWSR1-FLI1 ChIP-seq binding sites coincident with the GGAA microsatellites described in Orth et al. [30]. We found that no FEZF1/EWSR1-FLI1 regulated genes had a peak of 10 Kb upstream TSS and only two genes (FEZF1 and PHOSPHO1) had a peak 10 Kb downstream TSS. These results suggest that EWSR1-FLI1 does not directly regulate the 67 common genes regulated by FEZF1 / EWSR1-FLI1, but rather through FEZF1.

## 4. Discussion

Ewing sarcoma is an aggressive tumor that arises mainly in the bones of children and young adults. It is characterized by pathognomonic chromosomal translocations that produce chimeric transcription factors, for example, EWSR1-FLI1. Since the discovery of the fusion genes characteristic of Ewing sarcomas, there has been a strong interest in identifying the genes regulated by these chimeric transcription factors and analyzing their contribution to the development of Ewing sarcoma. Overall, these fusion genes induce a gene expression profile that increases proliferation and blocks cell differentiation. While this global pattern seems to be well-defined thanks to a multitude of functional genomics studies, it is still necessary to identify and functionally analyze each of the genes regulated by these fusion genes to further understand the molecular basis underlying this tumor development. On this occasion, we have focused our interest on the identification of transcription factors and/or transcriptional co-regulators that are also targeted genes of EWSR-FLI1. Using the widely used A673/TR/shEF cell model system, we showed that EWSR1-FLI1 significantly regulates the expression of more than 200 transcription factors or transcriptional regulators, accounting for approximately 15% of the known transcription factors and co-regulators. These results suggest that EWSR1-FLI1 exerts an important part of its regulatory activity through the regulation of other transcription factors that, in turn, regulate its own set of target genes. The result is a complex network of interactions between transcription factors and target genes that ultimately gives rise to the characteristic gene expression pattern of Ewing sarcoma.

The transcription factor that showed the most significant regulation by EWSR1-FLI1 was FEZF1. FEZF1 is a transcriptional regulator belonging to the C2H2 zinc finger family of transcription factors, involved in neurogenesis and, more specifically, in the maturation of olfactory sensory neurons during embryonic development [20,21,22,23,24]. FEZF1 was upregulated by EWSR1-FLI1 and was highly expressed in Ewing sarcoma cells when compared to other bone sarcomas and normal tissues. The fact that FEZF1 was expressed at high levels in all Ewing sarcoma cells tested, excepting the Ewing sarcoma cell line CADO-ES1 harboring an EWSR1-ERG fusion, suggest that FEZF1 can play a relevant role in Ewing sarcoma pathogenesis. The low expression of FEZF1 observed in CADO-ES1 cells cannot be attributed to the EWSR1-ERG fusion present in these cells since other cell lines with the same type of fusion expressed high levels of FEZF1, and its expression was significantly reduced when the expression of EWSR1-ERG was downregulated in other shRNA cell models (Figure S1). CADO-ES1 cells show some characteristics that are rarely seen in other Ewing sarcoma cell lines, as their ability to undergo chondrogenic differentiation in vivo xenograft models. The low expression of FEZF1 observed in CADO-ES1 cells can represent other characteristics that differentiate these cells from other “normal” Ewing sarcoma cells. To analyze the function of FEZF1 in Ewing sarcoma cells, we generated three Ewing sarcoma cell lines in which FEZF1 expression can be knocked down using a doxycycline-dependent shRNA system. Downregulation of FEZF1 expression in A673, SK-N-MC and SK-ES-1 Ewing sarcoma cells decreased cell proliferation and their ability to grow in clonogenic assays. As far as we know, this is the first time that a relationship between FEZF1 and Ewing sarcoma has been described and also one of the few times that FEZF1 has been studied in the cancer context. Lan et al. recently described that FEZF1 expression was associated with tumor relapse in cervical cancer patients and that FEZF1 knock-down in human cervical cancer cell lines reduced cell proliferation and cell migration by interaction with the Wnt pathway [25]. Meanwhile, Yu et al. described that FEZF1 was an independent biomarker to predict reduced survival in gliomas and that FEZF1 promoted proliferation, migration and invasion of glioma cells in vitro [43]. Interestingly, FEZF1 was shown to upregulate the expression of the oncogenic gene CDC25A, activating the PI3K/AKT pathway and promoting the malignant behavior of glioma stem cells [44]. The exact mechanism through which FEZF1 regulates proliferation in Ewing sarcoma cells is currently unknown, and therefore it will be important to determine in the future which pathways are involved. While the studies on FEZF1 and cancer are very scarce, the studies focusing on FEZF1-AS1 are much more numerous. FEZF1-AS1 is an lncRNA located near FEZF1, which is transcribed in the opposite orientation to FEZF1 and expressed at high levels in pancreatic cancer, colorectal cancer, lung adenocarcinoma and other human malignancies. FEZF1-AS1 has been associated with poor prognosis and has been shown to regulate proliferation, migration and invasion in various tumor cells (reviewed in [45]).

We have shown that FEZF1 and FEZF1-AS1 mRNA levels correlate strongly in Ewing sarcoma cells and that EWSR1-FLI1 knock-down drastically reduces the levels of FEZF1 and FEZF1-AS1 mRNAs, suggesting a coordinated regulation of both genes, at least in Ewing sarcoma cells. In the case of Ewing sarcoma, such coordinated regulation probably involves the binding of EWSR1-FLI1 to the long GGAA-microsatellite located in the exon 1 of *FEZF1*, as demonstrated from publicly available ChIP-seq datasets [3,30]. Since we are interested in analyzing the role of FEZF1 in Ewing sarcoma cells, we put special attention into designing a specific shRNA strategy to specifically knock-down FEZF1 mRNA without affecting the expression of FEZF1-AS1. Thus, we were able to downregulate the expression of FEZF1 without altering the expression of FEZF1-AS1. Downregulation of FEZF1 mRNA did not affect the expression of FEZF1-AS1, and thus, the effects observed upon FEZF1 knock-down should be associated exclusively with FEZF1. Thus, the effect observed on cell proliferation upon FEZF1 knock-down is attributable exclusively to the downregulation of FEZF1 levels. This observation is even more valuable if it is taken into account that in our cell models, levels of EWSR1-FLI1 remained high despite FEZF1 knock-down.

Despite Ewing sarcoma being described 100 years ago [6], the cell of origin of these tumors remains unknown, and many controversial origins have been proposed. One of the proposed hypotheses postulates that Ewing sarcoma tumors are from bone marrow-derived human mesenchymal cells. Supporting this hypothesis are the findings that ectopic expression of EWSR1-FLI1 in these cells is able to promote a transition to an Ewing sarcoma-like phenotype [46,47]. Another possibility is that these tumors arise from cells derived from the neural crest. In support of this, there is also some observational and experimental evidence. For example, some Ewing sarcoma tumors display an immature neural phenotype. Moreover, the Ewing sarcoma gene expression signature shows a reminiscent of neural lineages, and some Ewing sarcoma cells are able to display neural differentiation upon determined experimental conditions [8,9,10,11,12,13,15,48,49]. Finally, several studies suggest that EWSR1-FLI1 itself is able to determine the phenotype of Ewing sarcoma cells beyond the cell of origin. According to this hypothesis, EWSR1-FLI1 would be able to impose an expression signature that would determine the phenotype of Ewing sarcoma cells independently of the cell of origin. In other words, EWSR1-FLI1 would be able to “erase” the phenotype of the cell of origin and impose a new phenotype controlled by EWSR1-FLI1 [16]. The molecular mechanism involved in this reminiscent neural phenotype is largely unknown. Interestingly, we have shown that FEZF1 is able to regulate a neural-specific signature in Ewing sarcoma A673 cells by modulating the expression of genes involved in the formation of axons and vesicle trafficking in neurons, which is in agreement with the function of FEZF1 during embryonic development.

More importantly, some of the genes regulated by FEZF1 were regulated concomitantly by EWSR1-FLI1. Although the number of this set of genes regulated concomitantly by EWSR1-FLI1 and FEZF1 was small, some of these genes are particularly interesting. For example, ZIC5 (ZIC family member 5), OLFM3 (Olfactomedin 3) and SALL2 (Spalt-like transcription factor 2) were all downregulated upon EWSR1-FLI1 of FEZF1 knockdown (i.e., these genes correlated with EWSR1-FLI1 and FEZF1 expression). These genes were expressed at high levels in Ewing sarcoma cells according to public datasets [50] and are genes expressed in and related to the nervous system. Thus, ZIC5 is a putative transcriptional repressor involved in neural crest development, converting cells from an epidermal fate to a neural crest fate [51]. Interestingly, elevated expression of ZIC5 has been observed in various human cancers and may contribute to cancer progression [52,53,54]. OLFM3 belongs to the olfactomedin family. Although the exact function of these genes is largely unknown, their elevated expression in the brain suggests that they may have an essential role in nervous tissue [55]. Interestingly, other members of the olfactomedin family, OLFM1, were identified as one of the genes upregulated by EWSR1-FLI1 [15]. SALL2 is a transcription factor that plays a role in neurogenesis and eye development. In addition, SALL2 deregulation has been associated with cancer [56]. The fact that these genes were regulated in the same fashion upon EWSR1-FLI1 and FEZF1 knock-down suggests that they could be under the control (direct or indirect) of FEZF1.

## 5. Conclusions

In summary, we have shown that EWSR1-FLI1 regulates a significant number of transcription factors and transcriptional co-regulators. One of the most upregulated transcription factors was FEZF1, which was specifically expressed at high levels in Ewing sarcoma cell lines. Additionally, we have shown that the FEZF1 knock-down in three Ewing sarcoma cell lines reduced cell proliferation, suggesting that FEZF1 plays a role in the pathogenesis of this tumor. More interestingly, our results indicate that FEZF1 regulates, in Ewing sarcoma cells, a neural-specific gene signature that could be involved in maintaining the reminiscent neural phenotype observed in Ewing sarcoma. Our study highlights the importance of studying the contribution of transcription factors that are in turn regulated by EWSR1-FLI1 as a strategy to dissect the functional and genetic contribution of each of them to the pathogenesis of Ewing sarcoma.

## Figures and Tables

**Table 1:** Gene Ontology analysis of genes differentially regulated upon FEZF1 knock-down.

GO Cellular Component	Fold Enrichment	FDR	Downregulated Upon FEZF1 Knock-Down	Upregulated Upon FEZF1 Knock-Down
growth cone (GO:0030426)	4.99	0.048	UNC5C, CALM3, FMR1, MAP3K12, STMN3.	HAP1, NGFR, MYH14.
distal axon (GO:0150034)	4.57	0.016	UNC5C, CALM3, RAB5A, FMR1, MAP3K12, SYP, STMN3.	BLVRB, HAP1, VAT1, NGFR, MYH14.
axon (GO:0030424)	3.23	0.029	UNC5C, CALM3, SARM1, RAB5A, TENM3, ANK1, FMR1, MAP3K12, SYP, STMN3.	BLVRB, HAP1, VAT1, NGFR, MYH14, SEMA6A, ROGDI, SYT2.
transport vesicle membrane (GO:0030658)	4.93	0.036	SREBF1, CALM3, RAB5A, SYP, AFTPH.	VAT1, ARFGEF3, CEACAM1, SYT2.
transport vesicle (GO:0030133)	3.65	0.047	SREBF1, CALM3, RAB5A, RAB12, VGF, SYP, AFTPH, SYT2.	HAP1, VAT1, ARFGEF3, ROGDI, CEACAM1.

## Data Availability

The data presented in this study have been deposited in NCBI’s Gene Expression Omnibus (GEO) [57] and are accessible through GEO series accession number GSE184679 [58].

## Supplementary Materials

The following are available online at https://www.mdpi.com/article/10.3390/cancers13225668/s1, Figure S1: (A) FEZF1 mRNA expression in Ewing sarcoma cell lines analyzed in Orth et al. [30] (microarray data). (B) FEZF1 downregulation upon EWSR1-FLI1 or EWSR1-ERG knockdown. (C) EWSR1-FLI1 or EWSR1-ERG binding sites on FEZF1 GGAA-microsatellite, Figure S2: Representative examples of EWSR1-FLI1 binding sites detected by ChIP-seq, Figure S3: (A) Integrative Genome Visualizer (IGV) screenshot covering the FEZF1/FEZF1-AS1 genomic region. (B) FEZF1-AS1 mRNA expression (RT-qPCR) in cells A673/TR/shEF stimulated with doxycycline, Figure S4: Uncropped WB original images, Table S1: 1674 transcription factors that belong to the Gene Ontology term “DNA binding transcription factor activity” (GO:0003700), Table S2: Transcription factors/(co)regulators downregulated upon EWSR1-FLI1 knockdown in A673/TR/shEF cells, Table S3: Transcription factors/(co)regulators upregulated upon EWSR1-FLI1 knockdown in A673/TR/shEF cells, Table S4: Genes differentially regulated upon FEZF1 knockdown in A673/TR/shFEZF1 cells, Table S5: GO overrepresentation test.
