# Supplementary material for: The Transcription Factor FEZF1, a Direct Target of EWSR1-FLI1 in Ewing Sarcoma Cells, Regulates the Expression of Neural-Specific Genes
Source: Cancers (Basel). 2021 Nov 12;13(22):5668. doi: 10.3390/cancers13225668 (PMC8616448; doi:10.3390/cancers13225668)
Supplement: Supplementary file 1 [file cancers-13-05668-s001.zip › cancers-1422052-supplementary.pdf]

cancers

MDPI

Supplementary Materials: The Transcription Factor *FEZF1*, a Direct Target of *EWSR1-FLI1* in Ewing Sarcoma Cells, Regulates the Expression of Neural-Specific Genes

Laura García-García, Enrique Fernández-Tabanera, Saint T. Cervera, Raquel M. Melero-Fernández de Mera, Santiago Josa, Laura González-González, Carlos Rodríguez-Martín, Thomas G.P. Grünewald and Javier Alonso

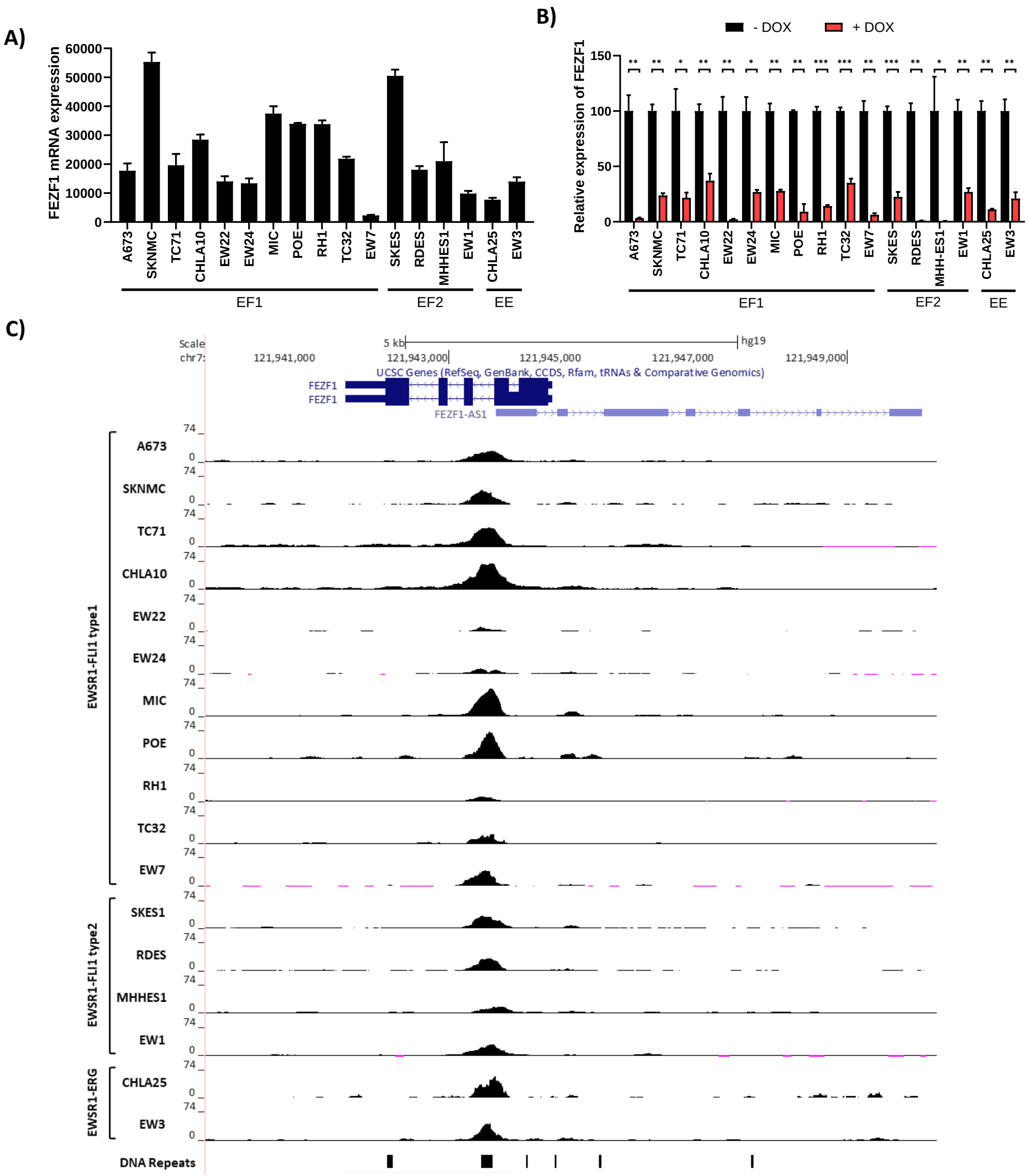

Figure S1. (**A**) FEZF1 mRNA expression in Ewing sarcoma cell lines analyzed in Orth et al. [29] (microarray data) (EF1, EWSR1-FLI1 type 1; EF2, EWSR1-FLI1 type 2; EE, EWSR1-ERG). (**B**) FEZF1 downregulation upon EWSR1-FLI1 or EWSR1-ERG knockdown. All cells showed a significant downregulation of FEZF1 upon EWSR1-FLI1/ERG silencing (doxycycline inducible shRNA cell models) (*P<0.05, **P<0.01, ***P<0.001, Student's T Test). (**C**) EWSR1-FLI1 or EWSR1-ERG binding sites on FEZF1 GGAA-microsatellite, as detected by ChIPseq analysis in Ewing sarcoma cell lines. All data extracted from publicly available datasets (GEO dataset GSE176400, Orth et al.).

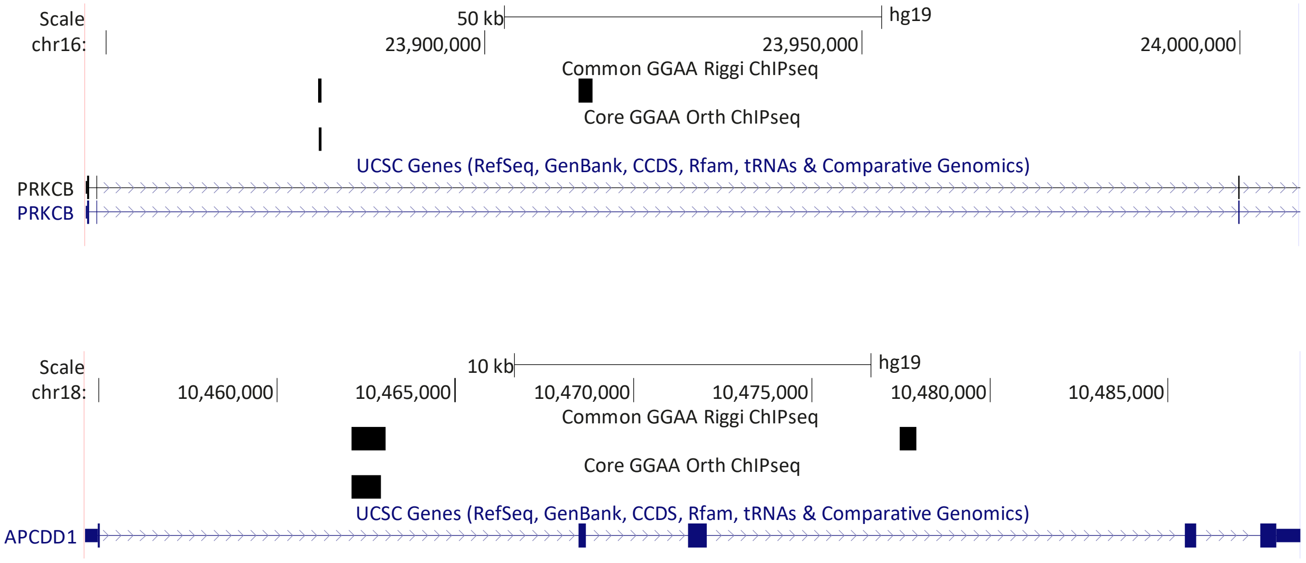

Figure S2. Representative examples of EWSR1-FLI1 binding sites detected by ChIP-seq.

Representative examples of EWSR1-FLI1 binding sites detected by ChIP-seq (Riggi et al., 2014 [3]; Orth et al., 2021 [29]) located at intronic sequences of two Ewing Sarcoma related genes (PRKCB and APCDD1). Straight blue line indicates introns, blue boxes indicate exons. EWSR1-FLI1 ChIP-seq peak tracks: “Common GGAA Riggi ChIPseq” are the EWSR1-FLI1 binding sites common to A673 and SKNMC cell lines from Riggi et al., 2014 [3]; “Core GGAA Orth ChIPseq” are the core EWSR1-FLI1 binding sites common to Ewing sarcoma cell lines analyzed in Orth et al., 2021 [29].

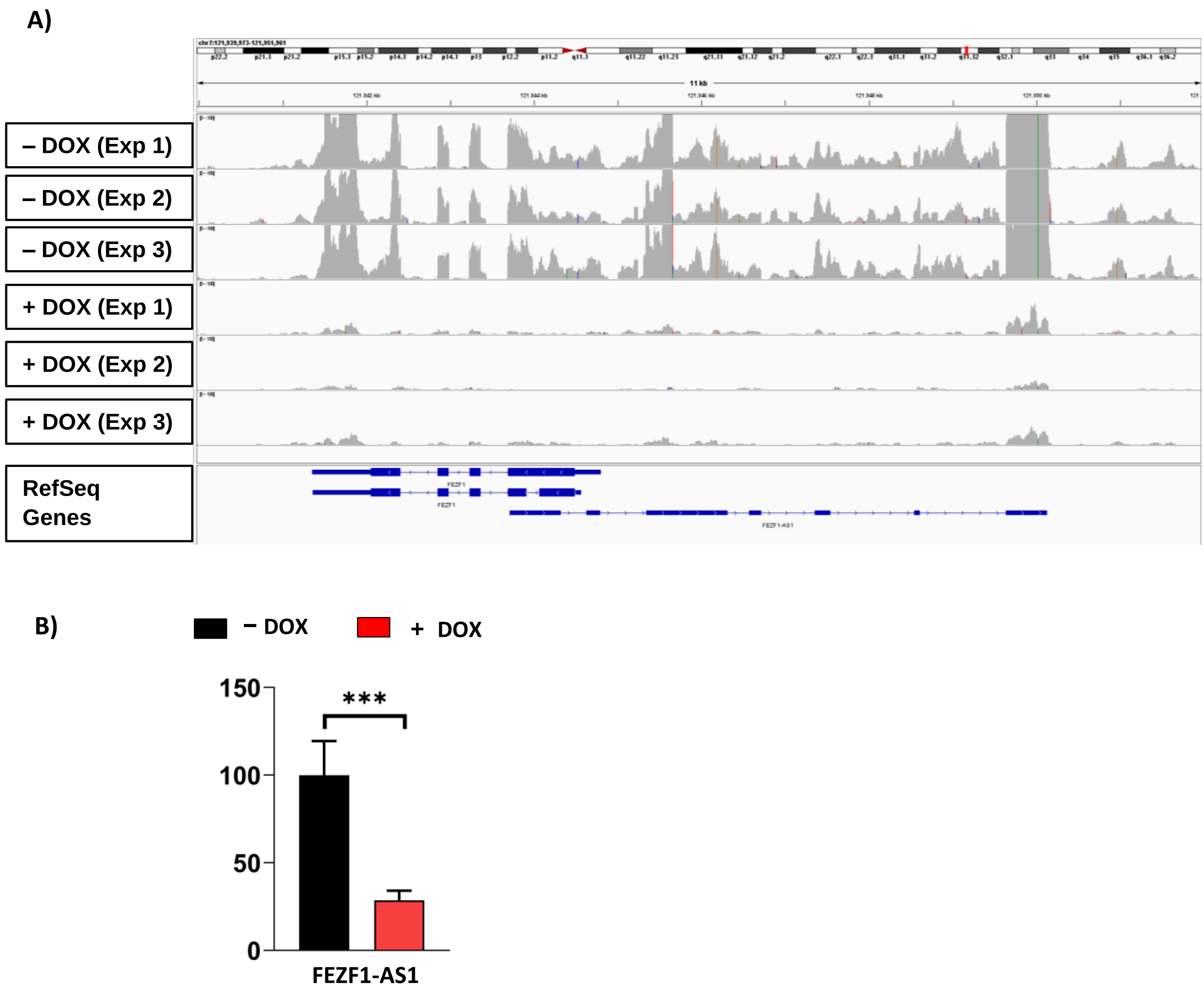

Figure S3. (**A**) Integrative Genome Visualizer (IGV) screenshot covering the FEZF1/FEZF1-AS1 genomic region, showing the read coverage in three independent RNAseq experiments performed with A673/TR/shEF cells in the absence (-DOX; EWSR1-FLI1high) or presence (+ DOX; EWSR1-FLI1low) of doxycycline. The number of reads covering FEZF1 and FEZF1-AS1 mRNAs were notably reduced in cells treated with doxycycline (EWSR1-FLI1low) in comparison to control cells. Scale was set to 0-100 in all cases. (**B**) FEZF1-AS1 mRNA expression (RT-qPCR) in cells A673/TR/shEF stimulated with doxycycline. FEZF1-AS1 mRNA levels were reduced near 75% upon EWSR1-FLI1 knockdown (***P<0.001; Student's T Test).

Figure 2B

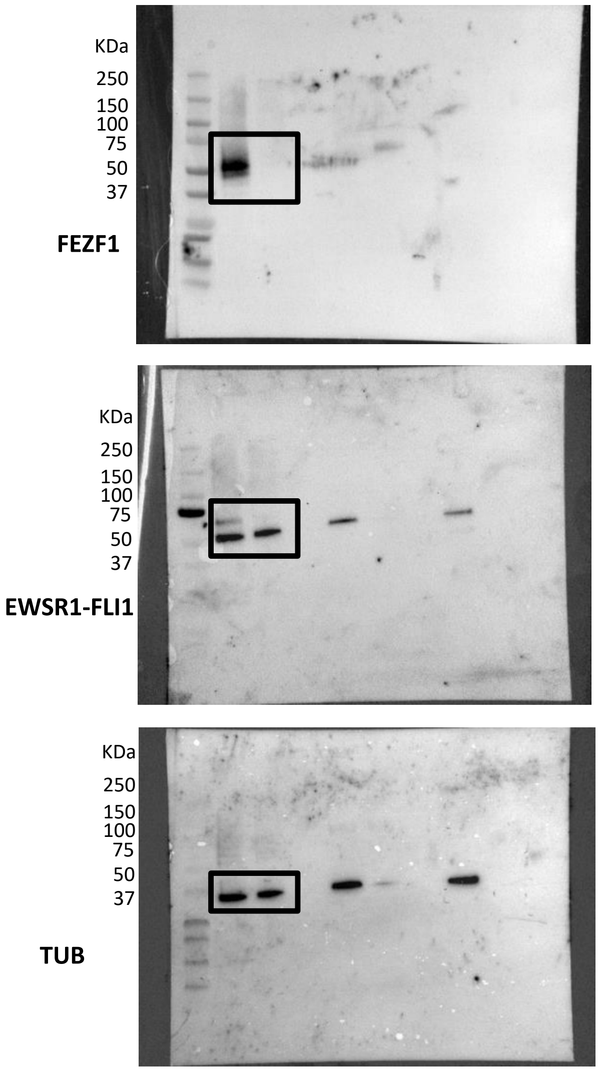

Figure 2E

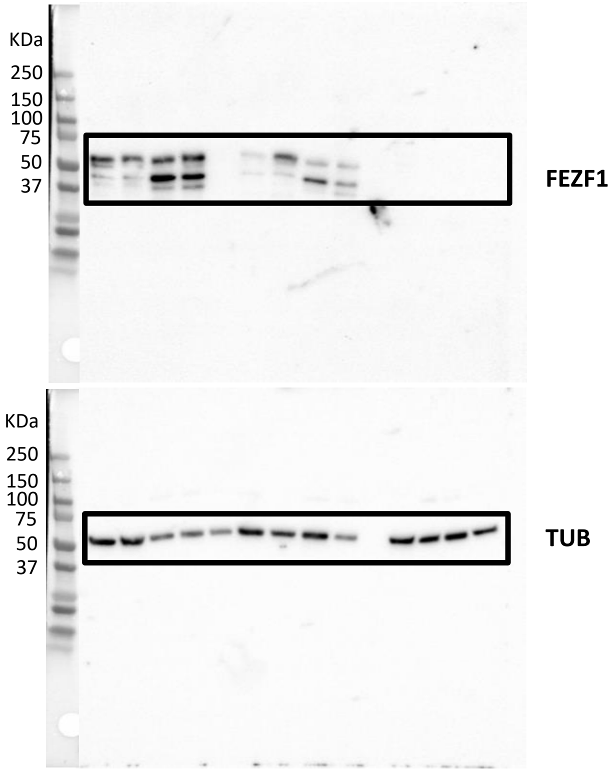

Figure 4B

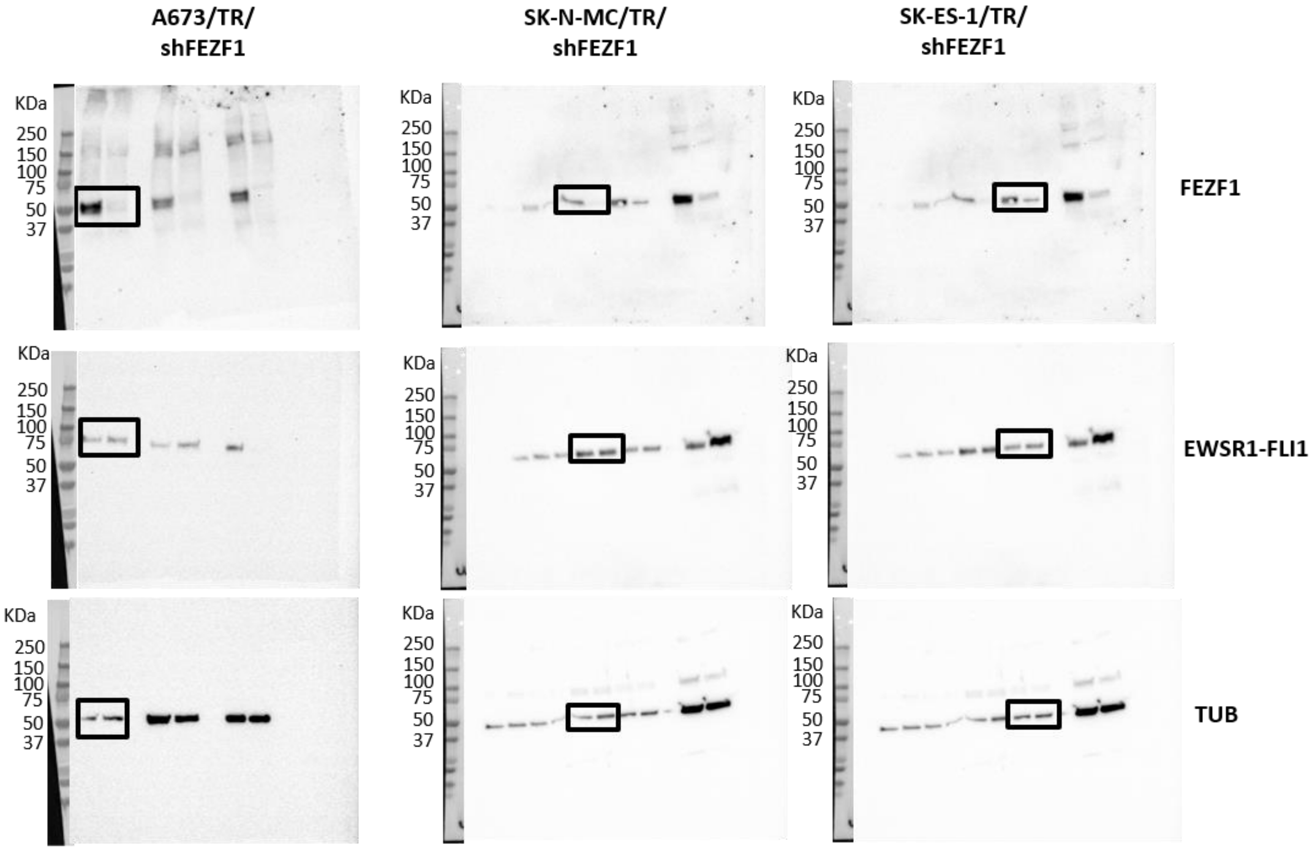

Figure S4. Uncropped WB original images.
